# Rising CO_2_ Interacts with Growth Light and Growth Rate to Alter Photosystem II Photoinactivation of the Coastal Diatom *Thalassiosira pseudonana*

**DOI:** 10.1371/journal.pone.0055562

**Published:** 2013-01-31

**Authors:** Gang Li, Douglas A. Campbell

**Affiliations:** 1 Biology Department, Mount Allison University, Sackville, New Brunswick, Canada; 2 Key Laboratory of Marine Bio-resources Sustainable Utilization, South China Sea Institute of Oceanology, CAS, Guangzhou, Guangdong, China; University of Hyderabad, India

## Abstract

We studied the interactive effects of pCO_2_ and growth light on the coastal marine diatom Thalassiosira pseudonana CCMP 1335 growing under ambient and expected end-of-the-century pCO_2_ (750 ppmv), and a range of growth light from 30 to 380 µmol photons·m^−2^·s^−1^. Elevated pCO_2_ significantly stimulated the growth of T. pseudonana under sub-saturating growth light, but not under saturating to super-saturating growth light. Under ambient pCO_2_ susceptibility to photoinactivation of photosystem II (σ_i_) increased with increasing growth rate, but cells growing under elevated pCO_2_ showed no dependence between growth rate and σ_i_, so under high growth light cells under elevated pCO_2_ were less susceptible to photoinactivation of photosystem II, and thus incurred a lower running cost to maintain photosystem II function. Growth light altered the contents of RbcL (RUBISCO) and PsaC (PSI) protein subunits, and the ratios among the subunits, but there were only limited effects on these and other protein pools between cells grown under ambient and elevated pCO_2_.

## Introduction

Atmospheric carbon dioxide (CO_2_) is expected to rise from current levels of ∼390 parts per million (ppm) to 700–1000 ppm by the end of this century, beyond the levels of the past 800 kyr of glacial-interglacial periods [1]. The dissolution of additional atmospheric CO_2_ into seawater alters the inorganic carbon buffer system by increasing the pCO_2_ and decreasing the pH [1], [2], potentially perturbing the physiological processes of marine phytoplankton including growth, photosynthesis and calcification [3], [4]. The elevated CO_2_ down-regulates the carbon concentrating mechanisms (CCMs) of phytoplankton, in particular in diatoms [5], [6]. Savings from this down-regulation are likely to allow compensatory increases in other processes such as phytoplankton growth [7]–[9], productivity [10] or synthesis of N-containing enzymes or cofactors [11]. These metabolic re-allocations could drive alterations in competitive interactions and niche boundaries among phytoplankton [12] and ultimately changes in community species compositions [13]–[16]. Rising CO_2_ caused decreases in the growth and productivity of monoculture or natural cell-assemblies under high solar irradiance, by increasing light stress and photorespiration [7], [16], [17]. Elevated pCO_2_ also decreased productivity of the diatom Thalassiosira pseudonana by increasing dark respiration [18]. In contrast, elevated CO_2_ had insignificant effects on the photophysiology of the marine diatom Chaetoceros brevis from the Antarctic Ocean [19], or on natural phytoplankton communities from the Derwent River estuary [20] or the Equatorial Pacific Ocean [21]. Such divergent responses to elevated CO_2_ complicate the implications of rising CO_2_ for marine phytoplankton.

In parallel with rising CO_2_, ocean temperature is increasing, which may increase stratification and decrease the upper-mixed-layer depth, thus exposing phytoplankton cells to higher mean light intensities [17]. Available light is a key factor for phytoplankton. Low light increases accessory pigmentation, which elevates the likelihood of photon capture [22]; it leads also to an increase in total proteins [23]. Concomitantly light drives photoinactivation of phytoplankton Photosystem II reaction centers through damaging key protein subunits, particularly PsbA, when can lead to photoinhibition or cell death if photoinactivation outruns counter-acting repair processes [24], [25].

Diatoms are a biogeochemically important group of marine phytoplankton. They contribute up to ∼40% of marine primary production and show relatively high carbon sequestration into the deep oceans because their silica frustules enhance their sinking rates [26], [27]. Diatoms often dominate in well-mixed coastal or estuarine waters where steep attenuation of light results in fast light fluctuations for cells mixing through the water column. Diatoms succeed under variable light through their high plasticity in photoacclimation capacity and quantum-to-biomass conversion rate [28] as well as their photoprotection mechanisms [29], [30]. Diatoms as a group [31], [32] have lower susceptibility to photoinactivation of PSII than do other phytoplankton [33]–[35]. This lowers the diatom cost-of-growth, particularly under fluctuating light, since they do not need to allocate as much protein metabolic capacity [25], [36] to counter PSII photoinactivation. Irradiance and elevated CO_2_ have interactive influences on diatom physiology which may contribute to controlling future community structures [15]–[19], [37].

In this paper we determined the interactive effects of elevated pCO_2_ and growth light on the photophysiology of the model diatom Thalassiosira pseudonana CCMP 1335 [38], [39], which is derived from a coastal environment, where light [40] and pCO_2_ [41] already vary widely.

## Materials and Methods

### Culture protocol

The coastal centric diatom, Thalassiosira pseudonana (Hustedt) Halse et Heimdal obtained from the Provasoli-Guillard National Center of Marine Phytoplankton (CCMP 1335) was cultured in 2 cm thick cuvettes (450 ml volume) of FMT-150 photobioreactors (Photon Systems Instruments, Drasov, Czech Republic) at 18°C in enriched artificial seawater (EASW) prepared according to [42], except with 54.5 µM Si and 0.82 µM Sr to limit precipitation during autoclaving. Cultures were gently mixed by a curtain of bubbles emitted from four apertures across the cuvette bottom with air at either ambient (∼390 ppmv) or elevated (∼750 ppmv) pCO_2_. Outdoor air was used for the ambient CO_2_ treatment whereas the elevated CO_2_ treatment was achieved by mixing 99.99% CO_2_ with zero-CO_2_ air using mass flow controllers (16 series, Qubit Systems, Kingston, Canada). Before bubbling into the bioreactor culture cuvette, the air streams were filtered through a 0.2 µm micro-filter and bubbled through sterile distilled water for humidification. The growth of T. pseudonana was not notably disrupted by this bubbling [43] although we earlier noted disruption of growth and cell breakage of larger diatoms using similar bubbling streams in these bioreactors (A. McCarthy & D. Campbell, unpub.).

Continuous growth light was measured with a microspherical quantum sensor (US-SQS, Waltz, Germany). The light intensities in culture vessels filled with seawater were set to 30, 80, 160, 240 or 380 µmol photons·m^−2^·s^−1^, provided by a panel of blue LED that cover the entire rear face of the cuvette. These light levels approximate a range from near the bottom of the euphotic zone at 1.5% of surface sunlight, up to the upper 35% of the euphotic zone in marine ecosystems.

### Carbon system analyses

During the growth period, we continuously monitored the pH in culture replicates in each of the three photobioreactors using an InPro 325× glass electrode (Mettler-Toledo) that was calibrated before setting up the culture systems with NBS buffers of pH 7.0 and 10.0 (Sigma-Aldrich). At the end of growth of each culture replicate, we measured dissolved inorganic carbon (DIC) with a CO_2_ analyzer (S151, Qubit systems, Kingston, Canada) [44]. We calculated total alkalinity, bicarbonate (HCO_3_^−^), carbonate (CO_3_^2−^) and pCO_2_ on the base of the measured temperature (18°C), salinity (35 g L^−1^), pH, DIC and the phosphate (21 µmol L^−1^) or silica (52.5 µmol L^−1^) contents of the prepared EASW using the CO2SYS program [45], with NIST scale constants from [46] and K1, K2 from [47] to calculate total alkalinity.

### Growth rate

We obtained a preliminary growth curve by operating the bioreactors in batch mode to determine a cell density set-point for turbidostat mode that maintained a stable pH, indicating stable DIC status, and a detectable F_O_ fluorescence signal from the diatoms, monitored continuously using the onboard sensor in the bioreactors. For each of the ten different combinations of light and pCO_2_, we then grew 3 or 4 replicate cultures by re-inoculating cells after cleaning and autoclaving the photobioreactor cuvettes.

**Figure 1:**
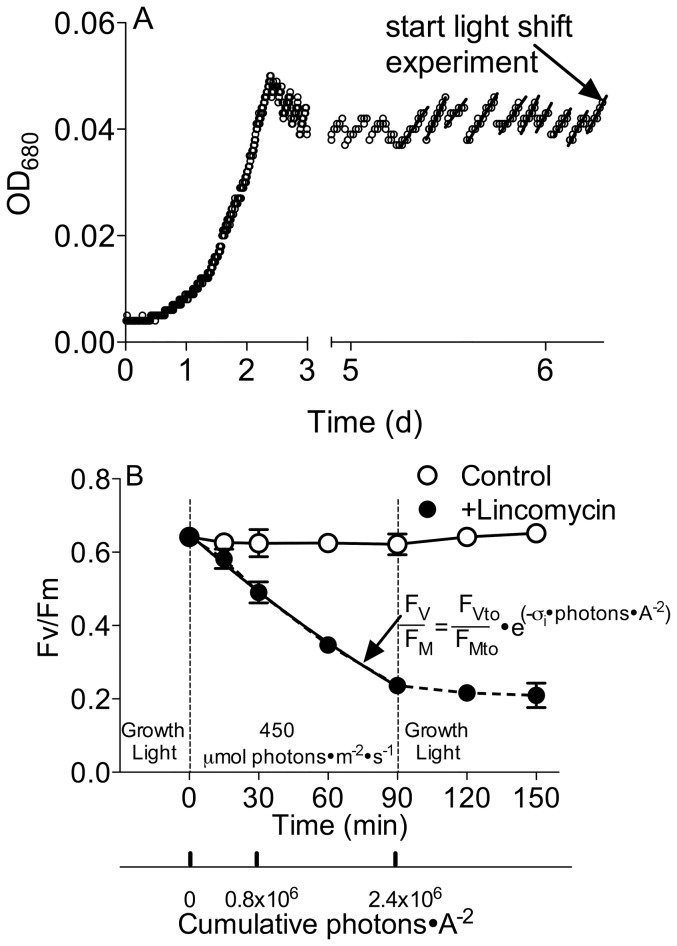
Measuring growth rate and Photosystem II Photoinactivation. A) Turbidostat culture cell density was maintained at set-point by automatic dilutions activated by a sensor tracking optical density at 680 nm. The last 10 cycles of dilution before starting the light-shift experiment were used to calculate the growth rate (μ, d^−1^) by fitting the increase in OD_680_ versus time with an exponential curve for each dilution cycle. This example growth curve was taken from a culture growing at 240 µmol photons·m^−2^·s^−1^, under bubbling with elevated pCO_2_ of 750 ppmv. B) PSII photochemical yield during and after an upward light shift. Cells were shifted from the culture growth light (240 µmol photons·m^−2^·s^−1^ in this example; 30, 80, 160, 240 or 380 µmol photons·m^−2^·s^−1^ in other experiments) upward to 450 µmol photons·m^−2^·s^−1^ and then back to the culture growth light again. Susceptibility to photoinactivation (σ_i_, A^2^) was obtained by fitting an exponential decay curve to the decrease in F_V_/F_M_ versus cumulative photons, in the sub-culture in which PSII repair was blocked by lincomycin.

**Figure 2:**
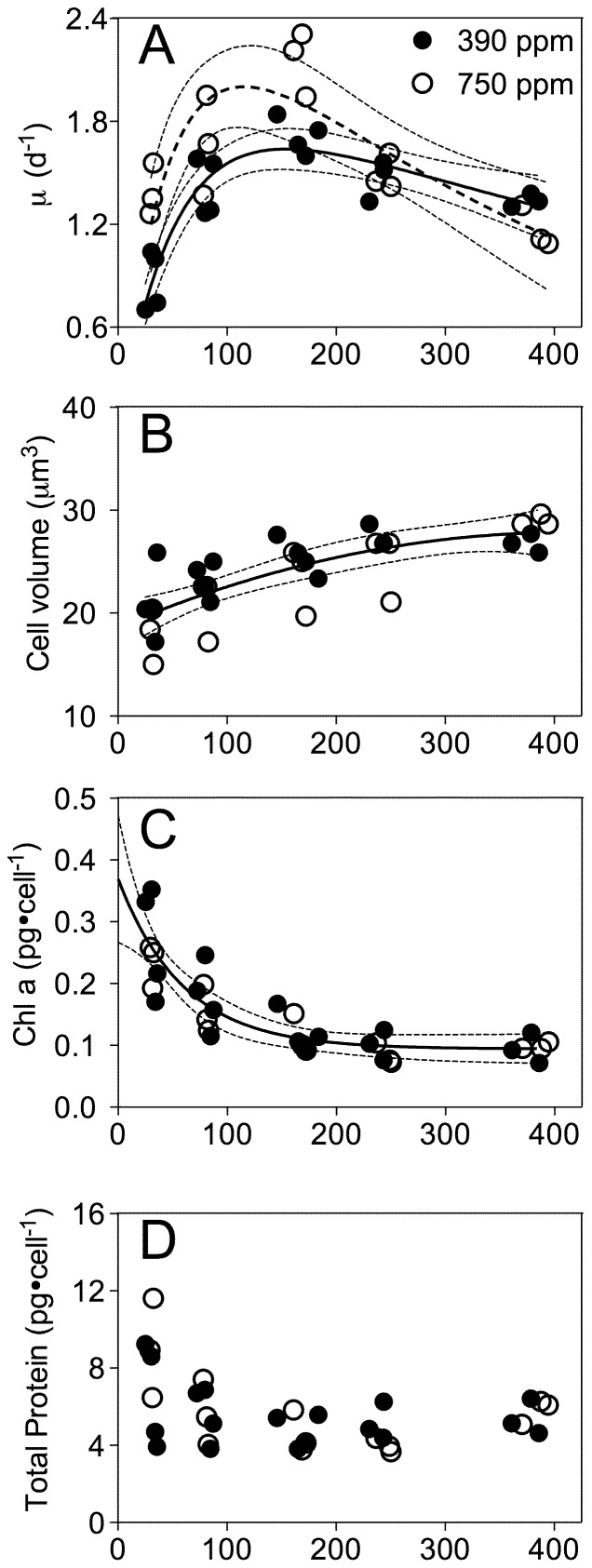
Growth rate, cell volume, Chl a and cellular protein versus growth light. A) Growth rate versus culture growth light under ambient or elevated pCO_2_. Solid line: Light response curve for growth of cultures at ambient pCO_2_. Dashed line: Light response curve for growth of cultures at elevated pCO_2_. Thin dotted lines: 95% confidence intervals on the fitted curves. B) Cell volume versus growth light under ambient or elevated pCO_2_. Solid line: Polynomial curve for pooled growth light response of cell volume for growth of cultures under the two CO_2_ treatments. Thin dotted lines: 95% confidence intervals on the fitted curve. C) Chlorophyll a versus culture growth light under ambient or elevated pCO_2_. Solid line: Polynomial curve for pooled growth light response for cultures from the two CO_2_ treatments. Thin dotted lines: 95% confidence intervals on the fitted curve. D) Total protein per cell versus culture growth light under ambient or elevated pCO_2_. E) Chl a:total protein versus culture growth light under ambient or elevated pCO_2_. Solid line: Polynomial curve for cultures from ambient pCO_2_. Dashed line: Polynomial curve for cultures from elevated pCO_2_. Thin dotted lines: 95% confidence intervals on the fitted curves.

We grew each experimental replicate culture from initial inoculation for 3–4 generations without dilution until the culture density reached the set-point (Fig. 1A) of basal fluorescence value (F_O_ = 280; 62±30 ng Chl a mL^−1^ in this study). We then entered turbidostat mode to maintain this cell density by activating a peristaltic pump when the F_O_ value reached the set-point (Fig. 1A) to dilute the 450 ml culture with a 10% volumetric addition of media delivered from a reservoir, which was continuously pre-bubbled with the air stream to pre-equilibrate the dissolved inorganic carbon system of the media before addition to the culture volume. 3.5 L of media in the reservoir supported about 9.5 cell generations of growth for each replicate culture under the set conditions of light and pCO_2_, before we harvested for the light-shift experiments and biochemical analyses described below. The temporal duration of each turbidostat run varied between 160 to 200 h, depending upon the achieved growth rate of the culture, which in turn varied with the applied growth light and pCO_2_. We calculated the specific growth rate for a given culture replicate (Fig. 1A; Fig. 2A) by using the onboard detector to monitor OD_680_, and then fitting the increase with time with an exponential growth function for the interval between each turbidostat 10% dilution cycle. We then averaged the exponential rates from the final 10 cycles of dilution to estimate the growth rate for the culture replicate. There were small variations in the light sources among the three bioreactor units we used, so we plotted specific growth rates and other parameters versus the actual light level applied to a given culture replicate, resulting in small offsets in data points along the X axes of plots (ex. Fig. 2A). Across the 10 combinations of growth light and pCO_2_ we grew a total of 33 separate turbidostat runs between December 2011 and August 2012, although experiment failures and volume limitations meant that not every analytical measurement was performed for every turbidostat run. At the end of the turbidostat growth period we measured the cell volume and cell suspension density with a Multisizer 3 Counter (Beckman Coulter Inc., USA).

### Frustule thickess

Samples were harvested for scanning electron microscopy (SEM) by collecting cells from approximately 15 mL of culture using a Millipore vacuum filtration apparatus containing a 25 mm diameter, 1 µm pore size polytetrafluoroethylene membrane (Sterlitech Corp., Kent, WA). The cells were washed with 250 mL distilled water, resuspended in 1 mL distilled water and cleaned in disposable 18×150 mm borosilicate culture tubes (Fisher Scientific, Ottawa, ON) by adding 10 mL each of concentrated sulfuric and nitric acid in a boiling water bath for 45 min. Samples were washed again with 250 mL distilled water in the filtration apparatus and resuspended in 5 mL distilled water. For SEM examination, 0.1–0.5 mL of the cleaned samples were deposited on 10×10 mm squares of phonograph record substrate and dried at 45°C. This substrate provided microscopic grooves with side walls tilted 45 degrees relative to normal and allowed viewing of fractured valves at this angle [48]. Substrates were attached to 32 mm diameter aluminum support stubs using colloidal graphite, coated with ca. 5 nm gold in a Hummer 6.2 sputtering unit (Anatech USA, Union City, CA) and examined using a JEOL JSM-5600 SEM (JEOL USA, Peabody, MA) operating at 10 kV and 8 mm working distance. Six images of fractured valves oriented so that the fracture was facing upslope and parallel to the wall of the groove were collected for each treatment. Valve thickness was measured on magnification calibrated digital image files using the program dmfMeasure [49]. Thicknesses were blind measured by stripping identifying information from the images, renaming the files with a randomly generated file name, and mixing them with images from at least three other treatments. Measurements were made in the centermost part of the fracture in a region where the break appeared to be as perpendicular through the cell wall as possible. Measured thicknesses were corrected for tilt and coating thickness. Once measurements for all treatments were complete, 30 images were randomly selected and measured again. Test-retest variability was less than 2.3% in all cases.

### Photoinactivation (σ_i_) determination

At the end of the turbidostat growth period we took a 280 mL culture sample from each replicate for determination of the functional absorption cross-section for photons driving PSII photoinactivation (σ_i_, A^2^ quanta^−1^) [31], [34], [50]. We divided the culture sample into two flasks, and supplemented one with a final concentration of 500 µg mL^−1^ lincomycin (Sigma-Aldrich) to inhibit chloroplast ribosome function [8], [31], [51] to block PSII repair. We placed both the flasks in the dark for 10 min to allow the antibiotic (if present) to penetrate into cells and inhibit ribosome function. We then shifted the flasks to 450 µmol photons m^−2^ s^−1^ blue light (LEE #183, Panavision; peak transmission at 455–479 nm and 50% transmission at 406–529 nm, approximating the growth light quality in the bioreactors). In this way, we aimed to assess the responses of cells acclimated to a range of light levels, to a shift to higher light simulating an upward mixing event to the upper region of the euphotic zone. After 15, 30, 60 and 90 min exposure to 450 µmol photons m^−2^ s^−1^, we took samples from both flasks for chlorophyll fluorescence measurements (Fig. 1B). After 90 min we returned the cultures to their culture growth light level and tracked any recovery over 30 and 60 min. We estimated the susceptibility of PSII to photoinactivation (σ_i_, A^2^ quanta^−1^) by fitting the exponential decrease of PSII photochemical yield (F_V_/F_M_) of the lincomycin-treated sample versus cumulative incident photons per area (Fig. 1B). To obtain F_V_/F_M_, a 2 mL sample from each culture replicate at each time point was dark adapted for 5 min in a temperature-controlled cuvette holder (18°C). A blue-green modulated measuring light (4 Hz; Xenon-PAM, Waltz, Effeltrich, Germany) was applied to measure F_O_, followed by a saturating light pulse (4,000 µmol photons m^−2^ s^−1^, 600 ms) to measure the dark-adapted maximal fluorescence (F_M_). F_V_/F_M_ was calculated as:
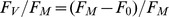



During the growth light recovery period we continued to monitor F_V_/F_M_ and if there was any increase in F_V_/F_M_ in the lincomycin treated cells we attributed the increase to slow relaxation of non-photochemical quenching, and used the amplitude of any relaxation to correct for the influence of non-photochemical quenching on changes in the measured F_V_/F_M_. Such corrections were small to negligible and had no substantive effect on the results.

### σ_PSII_ measurement

We measured the functional absorption cross-section for PSII photochemistry (σ_PSII_, A^2^ quanta^−1^) for blue light using a Fluorescence Induction and Relaxation fluorometer (FIRe, Satlantic, Halifax, Nova Scotia, Canada). Ahead of the light-shift experiment, we dark-acclimated a 2 mL sample from each culture replicate for 5 min. We then triggered an 80 µs single turnover flash (blue LED 455±2 nm) to saturate PSII photochemistry and drive fluorescence to a maximum. After this we exposed the sample to the relevant culture growth light (30, 80, 160, 240 or 380 µmol m^−2^ s^−1^) for 2 min. We then again triggered the single turnover saturating flash to obtain the absorption cross-section for PSII centers remaining open under the culture light level (σ′_PSII_). We then applied a high actinic light of 450 µmol m^−2^ s^−1^ to the samples for 2 min, followed by triggering the single turnover saturating flash again for σ″_PSII_ measurements of the absorption cross section for the remaining PSII centers still open under the high light treatment. The values of σ_PSII_ were extracted from the fluorescence rise curves using the FIReWORX software [52] with the flash irradiance calibration factors provided by Satlantic (Halifax, Canada).

Photochemical quenching to track the fraction of PSII centers open and ready for photochemistry under the growth light level for a given culture, was calculated following van Kooten and Snel (1990) [53] as:
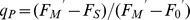



### Protein, Chl a and malondialdehyde measurements

Just prior to the high light shift experiments (T0 samples) or after the 90 min of high light treatment, both with or without lincomycin (T90 samples) we vacuum-filtered 50 or 30 mL of culture onto a binder-free Whatman GF/F glass fiber filter (25 mm in diameter), which was immediately flash frozen in liquid nitrogen and stored at −80°C until later analyses of protein and chlorophyll (50 mL filter) or malondialdehyde (30 mL filter).

Total protein was extracted from the frozen filters using the MPBio FastPrep®-24 with bead lysing matrix D (SKU 116913050) and 400 µL of 1× denaturing extraction buffer (0.1375 mol·L^−1^ TRIS buffer, 0.075 mol·L^−1^ LDS, 1.075 mol·L^−1^ glycerol, 0.5 mmol·L^−1^ EDTA, 0.1 mg·mL^−1^ Pefabloc) [54] for three cycles of 60 seconds at 6.5 m·s^−1^. Total protein concentration in the extracts was then determined using the Bio-Rad DC protein assay kit (500-0116) with known BGG standards. Molar levels of RbcL, PsbA, PsbD, PetC and PsaC in 1 µg total protein were then determined with quantitative immunoblotting [34], [51], [54].

For chlorophyll a (Chl a) measurement, 30 µL of protein extract was added to 470 µL 90% acetone (v/v) saturated with magnesium carbonate; after 15 min extraction in the dark at 4°C and 2 min centrifugation (13,000 g), we measured the absorbance of the supernatant at 664, 630 and 750 nm using a UV/VIS photospectrometer (UV-1800, Shimadzu, Japan). Chl a content was estimated following Jeffrey and Humphrey (1975) [55]:
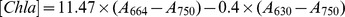



Malondialdehyde (MDA) content, an index of cumulative ROS toxicity, was determined with the thiobarbituric acid-reactive substance method [56], [57]. Cells on the filter were homogenized with 0.8 mL of 20% (w/v) trichloroacetic acid (TCA), followed by centrifugation at 13,000 g (10 min). After this, 0.35 mL of the supernatant and 0.35 mL of thiobarbituric acid reagent (0.5% in 20% TCA) was mixed, heated at 90°C for 30 min and cooled on ice. The absorbance of the mixture was measured at 532 and 600 nm with the extraction solvent as blank. Based on the A_532_–A_600_, MDA content was calculated by multiplying by the extinction coefficient of 6.45 µM cm^−1^.

### Carbon and nitrogen analyses

Prior to the light-shift experiment, we filtered 20 mL of turbidostat culture onto a pre-combusted (5 h, 450°C) Whatman GF/F glass fiber filter (13 mm in diameter), rinsed with 10 mL of 50 mmol L^−1^ HCl to remove inorganic carbon, dried at 55°C for 12 h and stored in a desiccator for later analyses. Contents of carbon and nitrogen were measured with a Vario EL III Elemental Analyzer (Elementar, Hanau, Germany).

### Data analysis

The response of growth rate and MDA content to culture growth light was fitted with the equation of Platt et al. (1980) [58]:
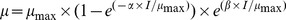
where: μ is growth rate (d^−1^) or MDA content (attomoles·cell^−1^) at the particular light level; μ_max_ is the maximum growth rate (d^−1^) or MDA content (attomoles·cell^−1^) derived from the curve fit; e is the base of natural logarithm; α is the initial quantum yield of growth or MDA content; I is the growth light (µmol photons·m^−2^·s^−1^) and β is an inhibition parameter.

We used ANOVA with Bonferroni post-tests (Prism 5, Graphpad Software) and comparisons of linear and non-linear curve fits to detect significant differences among the CO_2_ and culture-light treatments.

## Results

### Carbonate chemistry system

Carbonate chemistry parameters in our turbidostat cultures were representative of current ambient (∼390 ppmv) and the end of this century (∼750 ppmv), except that the estimated pCO_2_ level for cultures under the lowest growth light and elevated pCO_2_ treatment was significantly lower than in the cultures under higher growth lights (Table 1). As expected, the elevated pCO_2_ treatment increased DIC by 7.8%, HCO_3_^−^ by 13.6% and free CO_2_ in the medium by 117%, while pH decreased by 0.33 units and [CO_3_^2−^] decreased by 39.5%. The total alkalinity in the media remained unchanged between the ambient and elevated pCO_2_ treatments. The steady total alkalinity also shows that the differing rates of assimilation of ionic nitrogen and sulphur sources across the cultures growing at different rates did not significantly alter the media composition, which was instead dominated by the cycles of dilution with fresh media.

**Table 1:** Parameters of the seawater carbonate system of the cultures.

Growth light (µmol photons m^−2^ s^−1^)	Target pCO_2_ (ppm)	Dissolved Inorganic Carbon (µmol kg^−1^)	pH (NBS)	Total Alkalinity (µmol kg^−1^)	pCO_2_ (ppm)	CO_3_^2−^ (µmol kg^−1^)	HCO_3_^−^ (µmol kg^−1^)	CO_2_ (µmol kg^−1^)
31±3.4	390	1976±48	8.14±0.01	2302±56	312±3.7	1750±41	215±7.5	10.7±0.1
	750	2007±38	7.92±0.04	2213±62	551±50	1850±23	138±16	18.9±1.7
81±4.8	390	1866±20	8.18±0.07	2212±70	300±4.6	1632±15	209±13	9.1±1.6
	750	2047±15	7.85±0.01	2226±10	656±27	1903±17	121±2.9	22.5±0.9
167±12	390	1902±78	8.10±0.04	2196±107	332±16	1700±57	191±22	11.4±0.6
	750	2024±40	7.82±0.02	2186±33	706±50	1889±41	111±3.0	24.2±1.7
242±7.6	390	1908±93	8.10±0.05	2110±130	321±27	1630±67	184±29	11.0±1.0
	750	2064±63	7.86±0.06	2282±75	639±96	1916±64	126±15	22.0±3.3
380±12	390	1947±21	8.16±0.07	2286±57	295±53	1714±32	223±33	10.1±1.8
	750	2152±77	7.82±0.04	2320±92	745±45	2007±67	119±12	25.5±1.5

Dissolved inorganic carbon and pH were measured upon terminal sampling of turbidostat cultures grown under ambient- (390 ppm) and elevated- (750 ppm) CO_2_ treatments. Total alkalinity, pCO_2_, carbonate (CO_3_^2−^), bicarbonate (HCO_3_^−^) and free CO_2_ were calculated on the base of the temperature (18°C), salinity (35 g L^−1^), pH and concentrations of dissolved inorganic carbon, phosphate (21 µmol L^−1^) and silica (52.5 µmol L^−1^) using the software CO2SYS. n = 3, mean ±S.D.; n = 2 in treatments of 390, 750 pCO_2_ ppmv at the growth light of 31 µmol photons·m^−2^·s^−1^; for these estimates we present mean ±1/2 range.

### Growth, cell volume, Chl a and cellular protein

Cell growth rate increased with culture growth light from low (30 µmol photons·m^−2^·s^−1^) to moderate levels (160 µmol photons·m^−2^·s^−1^), but then decreased under higher light (240, 380 µmol photons·m^−2^·s^−1^) (Fig. 2A). Under bubbling with ambient pCO_2_ Thalassiosira psuedonana CCMP 1335 achieved a μ_max_ of 2.15 d^−1^. Bubbling with elevated pCO_2_ significantly stimulated the growth rate across low to moderate light level, allowing cells to achieve a μ_max_ of 3.04 d^−1^. Note that these estimates for μ_max_ are from the curve fits, which include an inhibition term. The measured maximum achieved growth rates for the cultures were 1.8 d^−1^ for ambient pCO_2_ and 2.3 d^−1^ for elevated pCO_2_. Above the saturating light level elevated pCO_2_ did not stimulate growth rate (Fig. 2A). There was an increase in cell volume from 19 to 28 µm^3^ as the growth light increased, but there was significant scatter among replicates and no significant effect of pCO_2_ on cell volume (Fig. 2B). Frustule thickness varied from 52 to 80 nm and showed significant scatter among replicates but no significant trend with growth light nor with pCO_2_ (data not presented).

Chl a content decreased from about 0.22 to 0.08 pg·cell^−1^ as the growth light increased from low to moderate levels, with no significant effect of elevated CO_2_ (Fig. 2C). There were no significant effects of growth light nor pCO_2_ on total protein per cell, although cultures growing under low light showed more scatter among replicates (Fig. 2D). The mass ratio of Chl a: protein decreased from 0.036 to 0.016 with the increasing growth light at ambient pCO_2_ (Fig. 2E) and from 0.025 to 0.015 under elevated pCO_2_, so cultures growing under elevated pCO_2_ showed a lower Chl a: protein, but only under low growth light (p<0.05). Note that the mass ratios for Chl a: protein were calculated for the same extract from each culture, and thus the patterns of the ratio versus light and pCO_2_ can achieve statistical significance even though the unpaired chlorophyll and protein plots do not, possibly because of experimental variation in extraction efficiencies.

### Photophysiological parameters

**Figure 3:**
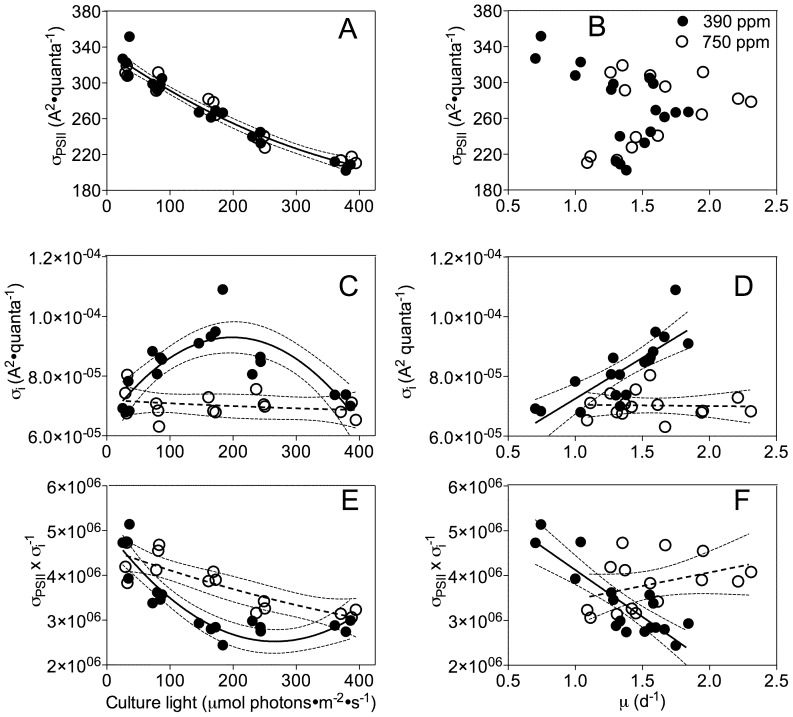
Photophysiology of cells grown under ambient and elevated CO2 versus growth light or growth rate. A) Functional absorption cross-section for Photosystem II photochemistry (σ_PSII_, A^2^·quanta^−1^) versus growth light. Solid line: Polynomial curve for pooled growth light response of σ_PSII_ under both ambient and elevated pCO_2_. Thin dotted lines: 95% confidence intervals on the fitted curve. B) Functional absorption cross-section for Photosystem II (σ_PSII_) versus growth rate. C) Functional cross section for photoinactivation of Photosystem II (σ_i_, A^2^·quanta^−1^) versus growth light. Solid line: Polynomial curve for growth light response of σ_i_ under ambient pCO_2_. Dashed line: Polynomial curve for growth light response of σ_i_ under elevated pCO_2_. Thin dotted lines: 95% confidence intervals on the fitted curves. D) Functional cross section for photoinactivation of Photosystem II (σ_i_) versus growth rate. Solid line: Linear regression for growth response of σ_i_ under ambient pCO_2_. Dashed line: Linear regression for growth response of σ_i_ under elevated pCO_2_. Thin dotted lines: 95% confidence intervals on the fitted curves. E) Ratio of σ_PSII_:σ_i_ versus growth light. Solid line: Polynomial curve for light response of σ_PSII_:σ_i_ under ambient pCO_2_. Dash line: Polynomial curve for growth light response of σ_PSII_:σ_i_ under elevated pCO_2_. Thin dotted lines: 95% confidence intervals on the fitted curves. F) Ratio of σ_PSII_:σ_i_ versus growth-rate. Solid line: Linear regression for growth response of σ_PSII_:σ_i_ under ambient pCO_2_. Dash line: Linear regression for growth response of σ_PSII_:σ_i_ under elevated pCO_2_. Thin dotted lines: 95% confidence intervals on the fitted curves.

The cultures showed differential growth-light and growth-rate dependent changes in photophysiology under ambient and elevated pCO_2_ (Fig. 3). The functional absorption cross-section that serves PSII photochemistry (σ_PSII_) decreased markedly from about 322 to 210 A^2^·quanta^−1^ with increasing growth light, with no significant effect of pCO_2_ (Fig. 3A). There was no pattern of σ_PSII_ with respect to culture growth rate (Fig. 3B), and so σ_PSII_ shows a clear pattern of acclimation to increasing culture growth light.

In marked contrast the functional absorption cross-section for incident photons driving PSII photoinactivation (σ_i_) increased from approximately 7.3×10^−5^ to 9.3×10^−5^ A^2^·quanta^−1^, as the growth light increased from 30 to 160 µmol photons·m^−2^·s^−1^ for cultures bubbled with ambient CO_2_, and then decreased back to 7×10^−5^ A^2^·quanta^−1^ as growth light increased further to 380 µmol photons·m^−2^·s^−1^ (Fig. 3C). Under elevated CO_2_ this curvilinear response to growth light disappeared and σ_i_ remained steady at ∼7×10^−5^ A^2^·quanta^−1^ (Fig. 3C). The unexpected curvilinear light response of σ_i_ is resolved by a plot of σ_i_ versus growth rate (Fig. 3D) which shows a positive, linear correlation for cultures growing under ambient pCO_2_ (R^2^ = 0.64, p<0.0001). Cultures grown under elevated pCO_2_ lose this correlation of susceptibility to photoinactivation with growth rate, and thus show a significantly lower σ_i_ under conditions of high growth rate (p<0.01) (Fig. 3D). Thus, under elevated pCO_2_ rapidly growing cells show a decrease in susceptibility to photoinactivation of PSII, compared to cells under ambient pCO_2_. In contrast, under slow growth elevated pCO_2_ can even provoke increased susceptibility to photoinactivation [8].

The ratio of σ_PSII_/σ_i_, a baseline estimate of photochemical return on investment per cycle of PSII repair, demonstrated both growth light and growth rate-dependent effects at both CO_2_ levels. σ_PSII_/σ_i_ in cultures bubbled with ambient pCO_2_ curvedly decreased from 4.5×10^−6^ to 2.8×10^−6^ as growth light increased from low to moderate levels. Cultures bubbled with elevated pCO_2_ showed a linear decrease in σ_PSII_/σ_i_ with growth light (Fig. 3E). At the moderate, growth-saturating light of 160 µmol photons·m^−2^·s^−1^, the σ_PSII_/σ_i_ in cultures bubbled with ambient pCO_2_ is about 70% of the value for cultures bubbled with elevated pCO_2_, indicating fewer rounds of photochemical charge separation per round of PSII repair under ambient CO_2_, where cells suffer more frequent photoinactivations of PSII under moderate light. At the fastest growth rates (Fig. 3F) cells under elevated pCO_2_, enjoy more potential photochemical return per round of PSII repair.

**Figure 4:**
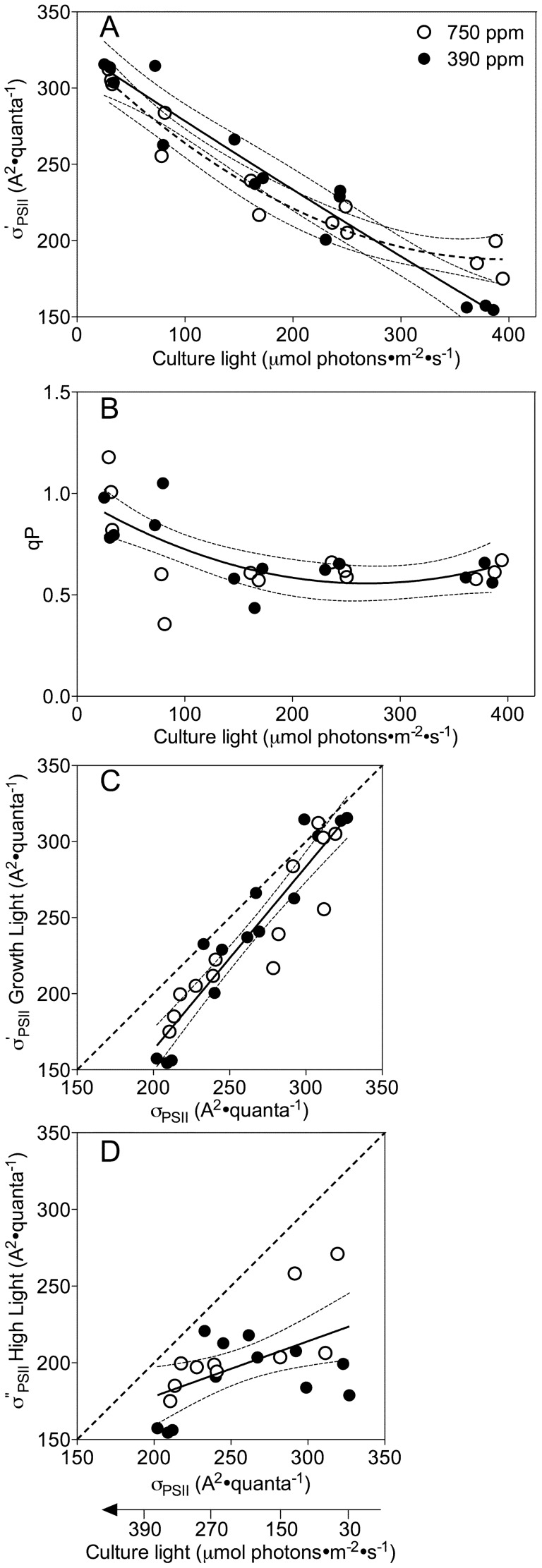
Effects of growth light and pCO2 on functional absorption cross-section for PSII photochemistry. A) Functional absorption cross-section for Photosystem II measured under culture light level (σ′_PSII_, A^2^·quanta^−1^) under ambient or elevated pCO_2_ plotted against the culture growth light (µmol photons·m^−2^·s^−1^). Solid line: Polynomial curve for growth light response of σ′_PSII_ under ambient pCO_2_. Dashed line: Polynomial curve for light response of σ′_PSII_ under elevated pCO_2_. Thin dotted lines: 95% confidence intervals on the fitted curves. B) Photochemical quenching (qP) versus growth light under ambient or elevated pCO_2_. Solid line: Polynomial curve for pooled growth light response for cultures from the two CO_2_ treatments. Thin dotted lines: 95% confidence intervals on the fitted curve. C) Functional absorption cross-section (σ′_PSII_) of the samples measured after 2 min of acclimation to culture-light, as a function of σ_PSII_ measured after 5 min dark-acclimation. Solid line: Linear regression of pooled response of cultures from ambient and elevated pCO_2_. Thin dotted lines: 95% confidence intervals on the fitted curve. Dotted diagonal line indicates 1∶1 ratio. D) Functional absorption cross-section (σ″_PSII_) of the samples measured after 2 min of acclimation to high-light of 450 µmol photons·m^−2^·s^−1^, as a function of σ_PSII_ measured after 5 min dark-acclimation. Solid line: Linear regression of pooled response of cultures from ambient and elevated pCO_2_. Thin dotted lines: 95% confidence intervals on the fitted curve. Dotted diagonal line indicates 1∶1 ratio.

The functional absorption cross-section for PSII photochemistry measured under growth light (σ′_PSII_) (Fig. 4A) shows a similar trend to σ_PSII_ measured from dark-acclimated cells (Fig. 3A), decreasing from about 311 to 156 A^2^·quanta^−1^ with the increasing growth light, with no significant effect of pCO_2_ except under the highest growth light of 380 µmol photons·m^−2^·s^−1^ (Fig. 4A). Photochemical quenching (qP) that tracks the fraction of PSII centers open and ready for photochemistry, decreased from 0.89 to 0.63 as the growth light increased from low to moderate levels, then remained steady under higher growth light levels, with no significant CO_2_ effects (Fig. 4B). When we plotted the growth light σ′_PSII_ versus σ_PSII_, all the points fell close to the 1∶1 dotted line (Fig. 4C), indicating limited net down-regulation of the antenna size serving PSII under culture growth light, compared to the antenna size from dark-acclimated cells. However, when we plotted the functional absorption cross-section (σ″_PSII_) measured under the high shift light (450 µmol photons·m^−2^·s^−1^) to the σ_PSII_, all the points fell far below the 1∶1 line, especially in the cultures from low growth light (Fig. 4D), showing a significant down-regulation of the functional antenna size under the excess light conditions as non-photochemical quenching is induced [59].

### Carbon and nitrogen

**Figure 5:**
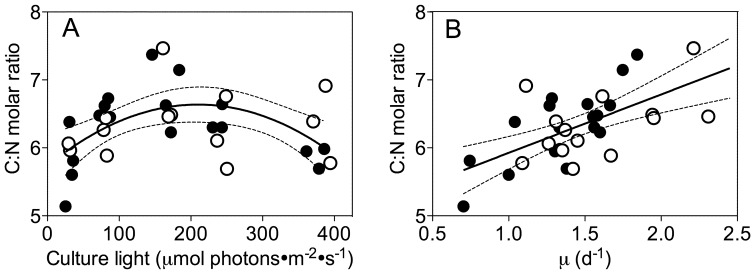
Molar ratio of C∶N versus growth light and growth rate. A) Molar ratio of carbon to nitrogen (C∶N) versus culture growth light. Solid line: Polynomial curve for pooled growth light response for cultures from ambient and elevated pCO_2_. Thin dotted lines: 95% confidence intervals on the fitted curves. B) Molar ratio of C∶N versus growth rate (μ, d^−1^). Solid line: Polynomial curve for pooled growth light response for cultures from ambient and elevated pCO_2_. Thin dotted lines: 95% confidence intervals on the fitted curves.

Mean contents of carbon or nitrogen were 0.65 and 0.11 pmol·cell^−1^, with no significant patterns in response to growth light nor pCO_2_ (data not presented). Under ambient pCO_2_ the molar ratio of C∶N varied from 5.1 to 7.4 and showed a curved response as the growth light increased from low to moderate levels, followed by a decrease under higher growth light levels (Fig. 5A); moreover, C∶N shows a linear increase with increasing growth rate (R^2^ = 0.37, p<0.05) (Fig. 5B) across both ambient and elevated pCO_2_ cultures.

### RbcL, PsbA, PsbD, PetC and PsaC

**Figure 6:**
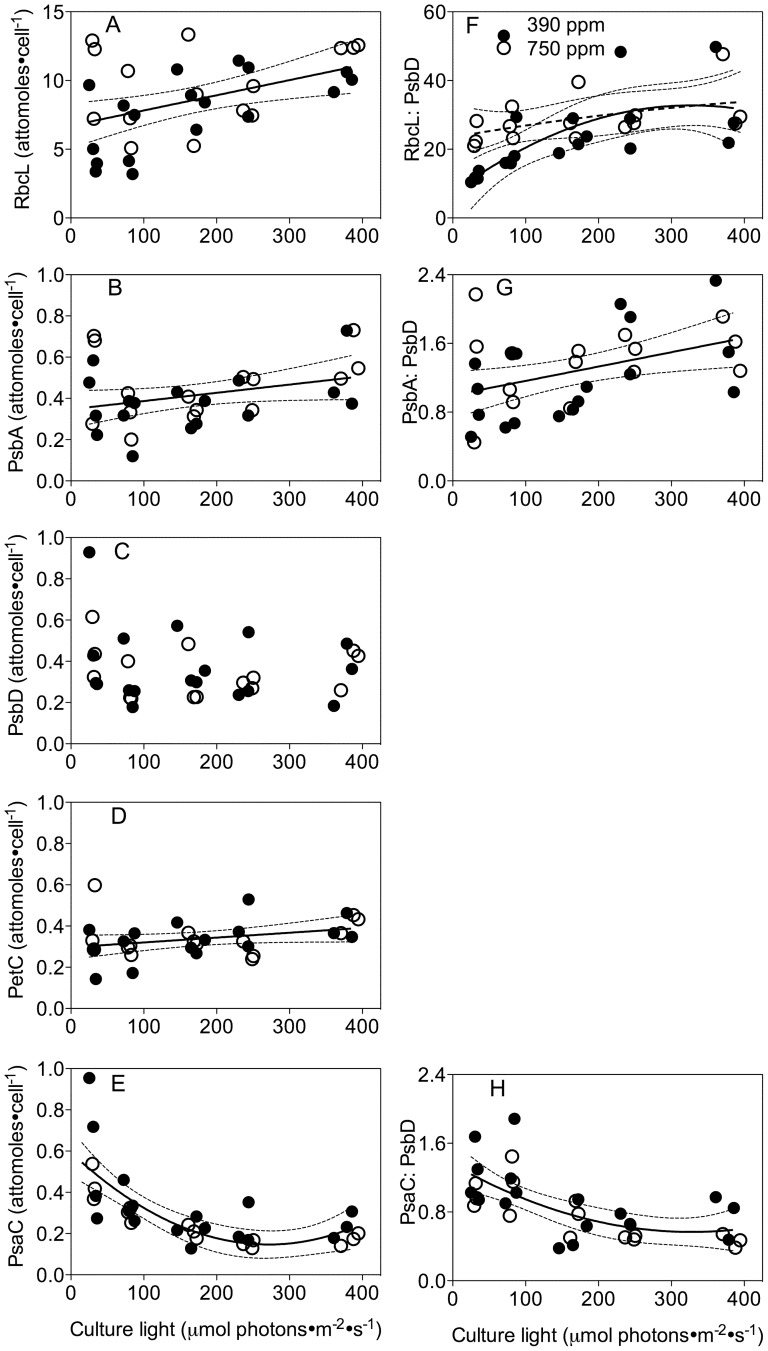
Representative protein subunits per cell for complexes mediating photosynthesis versus growth light. A) RbcL subunit from RUBISCO. Solid line: Linear regression for pooled growth light response for cultures from ambient and elevated pCO_2_. Thin dotted lines: 95% confidence intervals on the fitted curve. B) PsbA subunit from PSII versus growth light. C) PsbD subunit from PSII versus growth light. D) PetC subunit from cytochrome b_6_f complex versus growth light. E) PsaC subunit from PSI. Solid line: Polynomial curve for pooled growth light response for cultures from ambient and elevated pCO_2_. Thin dotted lines: 95% confidence intervals on the fitted curve. F) Ratio of RbcL: PsbD versus growth light, proxy for RUBISCO:PSII. Solid line: Polynomial curve for growth light response for cultures from ambient pCO_2_. Dash line: Polynomial curve for growth light response for cultures from elevated pCO_2_. Thin dotted lines: 95% confidence intervals on the fitted curves. G) Ratio of PsbA: PsbD versus growth light, proxy for PSII repair cycle. Solid line: Linear regression for pooled growth light response for cultures from ambient and elevated pCO_2_. Thin dotted lines: 95% confidence intervals on the fitted curve. H) Ratio of PsaC: PsbD versus growth light, proxy for PSI:PSII. Solid line: Polynomial curve for pooled growth light response for cultures from ambient and elevated pCO_2_. Thin dotted lines: 95% confidence intervals on the fitted curve.

Content of RbcL, the RUBISCO large subunit, varied from 3.2 to 11 attomoles·cell^−1^ and showed a modest positive correlation with increasing growth light (R^2^ = 0.21, p<0.05) (Fig. 6A). There were no significant effects of growth light nor pCO_2_ on the contents of PsbA (PSII subunit) (Fig. 6B), PsbD (PSII subunit) (Fig. 6C) nor PetC (Cytochrome b_6_f complex subunit) (Fig. 6D). However, PsaC (PSI subunit) declined significantly as growth light increased from low to moderate levels (Fig. 6E). Increasing growth light provoked significant increases in the ratios of RbcL∶PsbD and PsbA∶PsbD (Fig. 6F,G), but a decrease in the PsaC∶PsbD ratio (Fig. 6H). Overall we did not detect significant effects of pCO_2_ effects on protein subunit contents (Fig. 6), except under the growth light of 30 µmol photons·m^−2^·s^−1^ where the RbcL∶PsbD ratio was marginally higher in cultures bubbled with elevated pCO_2_ (p<0.05) (Fig. 6F).

### Malondialdehyde content

**Figure 7:**
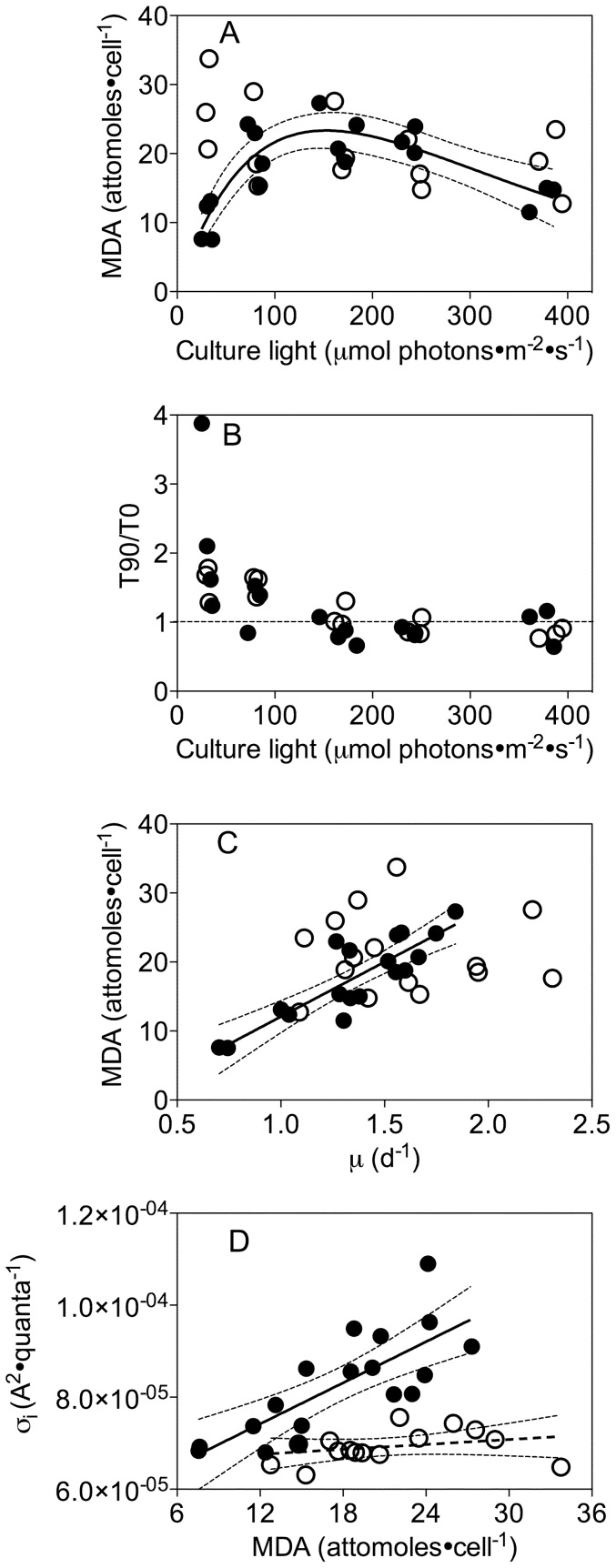
Malondialdehyde product of lipid peroxidation, growth rate and PSII photoinactivation. A) Malondialdehyde as a function of growth light. Solid line: Polynomial curve for malondialdehyde of cultures from ambient pCO_2_. Thin dotted lines: 95% confidence intervals on the fitted curve. B) Ratio of malondialdehyde measured at time 90 (T90) of a high light treatment to time 0 (T0) of control levels, versus growth light. Dotted horizontal line shows 1∶1 ratio of no change during the high light treatment period compared to the growth level. C) Malondialdehyde versus growth rate. Solid line: Linear regression of growth response of cultures from ambient pCO_2_. Thin dotted lines: 95% confidence intervals on the fitted curve. D) Functional cross section for photoinactivation of Photosystem II versus malondialdehyde. Solid line: Linear regression of σ_i_ versus malondialdehyde under ambient pCO_2_. Dashed line: Linear regression of σ_i_ versus malondialdehyde under elevated pCO_2_. Thin dotted lines: 95% confidence intervals on the fitted curves.

In cultures under ambient pCO_2_ malondialdehyde (MDA), a product of lipid peroxidation, curvedly increased from 10 to 23 attomoles·cell^−1^ as growth light increased from low to moderate levels, followed by a decrease to 14 attomoles·cell^−1^ under the highest growth light (Fig. 7A). Elevated pCO_2_ significantly increased the MDA content under low growth light (30 µmol photons·m^−2^·s^−1^), but not under higher growth light (Fig. 7A). The content of MDA was also increased after 90 min exposure of low-light-cultured cells (30 or 80 µmol photons·m^−2^·s^−1^) to 450 µmol photons·m^−2^·s^−1^ under both CO_2_ treatments (Fig. 7B). Furthermore, there was a positive correlation (R^2^ = 0.75, p<0.001) between the MDA content and growth rate under ambient pCO_2_, but not under elevated pCO_2_ (Fig. 7C), mirroring the correlation of σ_i_ with growth rate under ambient pCO_2_ (Fig. 3D,7D).

## Discussion

### Elevated pCO_2_ stimulates diatom growth under low to moderate light

A seawater inorganic carbon system that approximates conditions expected for the end of this century promoted the growth rate of a marine coastal diatom under sub-saturating growth light, but not under saturating to super-saturating growth light. The significant fertilization of elevated pCO_2_ on growth of the diatom under lower light (Fig. 2A) may be accounted for by an increased diffusive supply of free CO_2_ and bicarbonate concentrations, that could allow the diatom to down-regulate carbon concentrating mechanisms (CCM) [5], [6] and thereby save about 20% of the energy demanded by CCM under current ambient pCO_2_ [5], [60], [61]. The savings from this down-regulation can be re-allocated to support the increased growth rate (Fig. 2A). We see a hint of these resource re-allocations in the lower Chl: Protein ratio under low light and elevated pCO_2_ (Fig. 2E), but surprisingly not in our determinations of C∶N molar ratio (Fig. 5A) nor in representative subunits of major photosynthetic complexes (Fig. 6). Under higher growth lights savings from CCM down-regulation may become less significant relative to the costs of coping with excess light [7], [16], [18]. Furthermore with rapid growth under high light the increased demand for carbon assimilation may outweigh the influence of increased pCO_2_.

In our cultures the moderate light of 240 µmol photons·m^−2^·s^−1^ (Fig. 2A) was sufficient to provoke the onset of inhibition of growth, particularly under elevated pCO_2_. Cullen and Lewis (1988) [62], in contrast, achieved yet higher growth rates with T. pseudonana under light up to 2200 µmol photons·m^−2^·s^−1^. We suspect the difference arose because we grew the cultures under continuous illumination from narrow-band blue light LED sources, for consistency between our growth light spectra and our determinations of σ_PSII_ and σ_i_ for blue light. Blue light has a higher quantum yield for photoinactivation of PSII of diatoms [51] and plants [63] than do longer wavelengths, so our illumination provoked a higher rate of photoinactivation at a given light level than did the white light source of Cullen and Lewis (1988) [62].

### Elevated pCO_2_ alters the photophysiological properties of diatoms

When growing under current ambient pCO_2_ the coastal diatom showed a curvilinear relation between the susceptibility of their PSII to photoinactivation (σ_i_) and growth light (Fig. 3C). This surprising pattern resolved to a linear correlation between σ_i_ and growth rate (Fig. 3D). For determination of σ_i_ we elevated the treatment light to accelerate the rate of photoinactivation, to improve the accuracy of curve fits over reasonably short treatment times. As reviewed in Campbell &Tyystjärvi [50] σ_i_ is applicable across the range of growth lights, although at lower light the rate of photoinactivation (σ_i_×incident photons m^−2^ s^−1^) will be slower. The turbidostat cultures maintained a stable growth rate, with stable Photosystem II function, tracked with F_V_/F_M_, and stable PSII protein content (Fig. 6B,C). In this steady state situation each photoinactivation event is countered by the PSII repair cycle. Therefore, the susceptibility to photoinactivation is a direct measure of the metabolic cost the diatoms incur to maintain PSII function under a given growth light. Under ambient pCO_2_ the faster growing diatoms suffer a cost of increased susceptibility to photoinactivation and thus a proportionate increase in their metabolic cost to maintain photosynthesis. Under elevated pCO_2_ these patterns of σ_i_ disappear, so that at the very lowest growth rates diatoms under elevated pCO_2_ show similar (Fig. 3D) or even increased susceptibility to photoinactivation compared to current ambient pCO_2_ [8]. In contrast, under moderate to high growth rates the diatoms under elevated pCO_2_ enjoyed significantly lower susceptibility to photoinactivation. At the optimal growth light of 160 µmol photons·m^−2^·s^−1^ diatoms under elevated pCO_2_ show a ∼30% lower σ_i_, and thus incur a proportionately lower cost to maintain their PSII pool and photosynthetic capacity.

Note that these determinations of susceptibility to photoinactivation are for diatoms shifted to the moderately high light of 450 µmol photons·m^−2^·s^−1^ and that upon shifts to yet higher light additional factors interact with photoinactivation of PSII, including the catalytic capacity for ROS detoxification [64] and dumping of photosynthetic electrons back to oxygen [65]. Furthermore, our current study is of coastal diatoms under exponential growth and nutrient repletion and in some phytoplankton nutrient depletion strongly alters photophysiological responses [32], [66].

We tested multiple hypotheses as to the underlying mechanism(s) for the effects of growth rate and of elevated pCO_2_ upon photoinactivation. Based upon findings of Y. Wu & Z. Finkel (unpub. data) that elevated pCO_2_ lowers the silica content of diatoms, we measured the frustule thickness (data not presented) to determine if changes in frustule thickness might underlie the changes in susceptibility to photoinactivation, since the diatom frustule can screen harmful radiation [67]. We did not find any significant correlation of frustule thickness with growth light (data not presented), nor with growth rate, nor with pCO_2_, and so we did not find support for our initial hypothesis that pCO_2_ influenced cellular optics by altering frustule thickness. More subtle changes in frustule structure [68], density or pigment binding [67] could still potentially contribute to changes in diatom susceptibility to photoinactivation of PSII. We did capture whole cell spectra using an integrating cavity spectrophotometer but did not detect significant changes in cellular coloration under changing pCO_2_ (data not presented). The buffering influence of silicate upon pH changes under rising CO_2_ might also influence PSII susceptibility to photoinactivation of diatom by altering CCM operation [69], but we have no data on this topic. More generally we find a suggestive correlation between the diatom frustule and the low susceptibility of diatoms, as a group, to photoinactivation [31], when compared to other phytoplankton measured under comparable conditions including coccolithophores [8], [32], [33], picocyanobacteria [34], prasinophytes [35], *Bolidomonas* [70] and other taxa (Campbell et al. unpub.). Even here, however, specificities of diatom photosystem subunit composition [71] and metabolism [65] provide alternate explanations for the unusually low susceptibility of diatoms to photoinactivation of PSII.

σ_PSII_ showed a simple negative correlation with growth light, was not significantly altered by elevated pCO_2_ (Fig. 3A) and did not correlate with growth rate (Fig. 3B), thus showing straight forward light regulation of functional antenna size. Elevated pCO_2_ had no significant effects on the down regulation of σ″_PSII_ under excess light (Fig. 4D). Therefore the effects of light and pCO_2_ on σ_i_ (Fig. 3C,D) appear to arise outside and separately from acclimatory changes in the functional size and short-term regulation of the antenna serving PSII. Across a taxonomic panel of diatoms of widely differing size [31] we found negative correlations of both σ_PSII_ and σ_i_ with increasing cell biovolume, which is consistent with pigment packaging or self-screening effects in the larger cells [31], [72], [73]. We did see some increase in cell biovolume with increasing light in these experiments (Fig. 2B), but there was no significant influence of pCO_2_ on biovolume and thus the small changes in biovolume explained neither the effects of pCO_2_ nor growth light on σ_i_.

The change from ambient to elevated pCO_2_ did not significantly alter the pattern of PSII closure, measured as q_P_ with increasing growth light (Fig. 4B), nor was there an effect of pCO_2_ upon q_P_ measured under the higher treatment light (data not presented). Therefore we cannot attribute the pCO_2_ effect on susceptibility to photoinactivation to a simple change in excitation pressure on PSII [74].

We hypothesized that large re-allocations of nitrogen resources (Fig. 5) among the major complexes mediating photosynthesis might underlie the interactive effects of growth light and pCO_2_ on σ_i_. RbcL (RUBISCO, Fig. 6A) and PsaC (PSI, Fig. 6E) contents changed with growth light, but we found only modest effects of elevated pCO_2_ on the ratio between RbcL (RUBISCO) and PsbD (PSII subunit) and only in cells growing under low light (Fig. 6F). We did not detect any significant influence of pCO_2_ on the cellular contents of PSII subunits, so a bulk change in PSII content is unlikely to explain the patterns of σ_i_ with growth light and pCO_2_. We did detect a moderate increase in the PsbA∶PsbD ratio under increasing light, which may reflect increasing engagement of the PSII repair cycle and accumulation of intermediates in the overall PSII repair cycle [51], , but which does not explain the patterns of σ_i_ that we observe.

We analyzed the cellular content of MDA, an end product of lipid peroxidation produced when reactive oxygen stress damages biological membranes [56], [57], [76]. Increasing growth light and elevated pCO_2_ both altered MDA content of the diatoms (Fig. 7A). A shift from growth light to excess light of 450 µmol photons·m^−2^·s^−1^ provoked some additional accumulation of MDA, but only in cells growing under low light; cells growing under higher light showed no additional MDA accumulation (Fig. 7B) pointing to pre-induction of detoxification systems [64] sufficient to counter any additional ROS production during the high light shift. MDA also correlated with growth rate (Fig. 7C), particularly in cells growing under ambient pCO_2_. Carbon limitation has been observed to induce increased expression of ROS-removing enzymes [77], which can explain the lower MDA in cells growing under ambient than elevated pCO_2_, particularly under lower growth light (Fig. 7A). Intriguingly in cells under ambient pCO_2_ increasing susceptibility to photoinactivation correlates with increasing cellular content of MDA (Fig. 7D), suggesting that in cells suffering significant photoinactivation, lipid peroxidation occurs in parallel, possibly driven by common or over-lapping mechanisms [78]–[80]. In contrast, under elevated pCO_2_ cellular content of MDA varies widely depending upon growth conditions but σ_i_ does not, so growth under elevated pCO_2_ breaks the correlation between an index of ROS toxicity and cellular susceptibility to photoinactivation (Fig. 7D). In summary, our analyses to date have not yet uncovered a mechanism for the interactive effects pCO_2_, light and growth rate on σ_i_, but the effects are significant and represent biologically meaningful changes in the cost of maintaining the photosynthetic apparatus in this coastal diatom.

To maintain PSII function, every photoinactivation event must be countered by an expensive PSII repair process [25], [59], [75]. Assuming the similar cellular contents of PSII protein subunits under ambient and elevated pCO_2_ are proxies for similar overall pools of PSII, the lower σ_i_ under elevated pCO_2_ (Fig. 3D) implies a decreased metabolic burden to maintain a comparable PSII pool.

The ratio σ_PSII_/σ_i_ ratio tracks the delivery of excitons to drive PSII photochemistry relative to photoinactivation events and sets an upper boundary on the return-on-investment for maintaining PSII under a given growth light condition. Elevated pCO_2_ markedly alters this relation (Fig. 3E,F) so fast-growing diatoms under elevated pCO_2_ enjoy a significantly higher metabolic return from their investments into PSII.

The coastal strains of T. pseudonana that can dominate in the well mixed coastal or estuarine waters [27], [81] are well adapted to the sharply fluctuating light therein. Simultaneously, pH in these waters also varies widely, for example from 7.0 to 8.4 in the Pearl River estuary of the South China Sea, causing a drastic change in pCO_2_ of 380 to 4800 ppmv [41]. Thus coastal phytoplankton including strains of T. pseudonana must be well adapted to changing CO_2_ levels. T. pseudonana is also a bloom forming species whose own blooms can occasionally considerably change the seawater CO_2_ and light regimes.

Elevated pCO_2_ and growth light interactively affect the growth rates, photophysiological parameters and key protein levels of the coastal diatom T. pseudonana CCMP 1335. The elevated CO_2_ provokes a decreased susceptibility to photoinactivation of PSII when the growth rates are high (Fig. 3D). In nutrient-rich coastal or estuarine waters, the growth rate of natural phytoplankton assemblages including diatoms can reach as high as 2.5 d^−1^ [82]. Moreover, the diatoms in these waters must exploit variable light environments due to sharp attenuation of the light gradient and fast vertical mixing rates [40]. Decreased susceptibility to photoinactivation due to rising CO_2_ thus supplies T. pseudonana with a potential competitive advantage over other phytoplankton species in coastal or estuarine waters.
